# Insights into the physiology of *Chlorella vulgaris* cultivated in sweet sorghum bagasse hydrolysate for sustainable algal biomass and lipid production

**DOI:** 10.1038/s41598-021-86372-2

**Published:** 2021-03-24

**Authors:** Neha Arora, George P. Philippidis

**Affiliations:** Patel College of Global Sustainability, University of South Florida, 4202 E. Fowler Avenue, Tampa, FL 33620 USA

**Keywords:** Biochemistry, Biotechnology

## Abstract

Supplementing cultivation media with exogenous carbon sources enhances biomass and lipid production in microalgae. Utilization of renewable organic carbon from agricultural residues can potentially reduce the cost of algae cultivation, while enhancing sustainability. In the present investigation a medium was developed from sweet sorghum bagasse for cultivation of *Chlorella* under mixotrophic conditions. Using response surface methodology, the optimal values of critical process parameters were determined, namely inoculum cell density (O.D._750_) of 0.786, SSB hydrolysate content of the medium 25% v/v, and zero medium salinity, to achieve maximum lipid productivity of 120 mg/L/d. Enhanced biomass (3.44 g/L) and lipid content (40% of dry cell weight) were observed when the alga was cultivated in SSB hydrolysate under mixotrophic conditions compared to heterotrophic and photoautotrophic conditions. A time course investigation revealed distinct physiological responses in terms of cellular growth and biochemical composition of *C. vulgaris* cultivated in the various trophic modes. The determined carbohydrate and lipid profiles indicate that sugar addition to the cultivation medium boosts neutral lipid synthesis compared to structural lipids, suggesting that carbon flux is channeled towards triacylglycerol synthesis in the cells. Furthermore, the fatty acid profile of lipids extracted from mixotrophically grown cultures contained more saturated and monosaturated fatty acids, which are suitable for biofuel manufacturing. Scale-up studies in a photobioreactor using SSB hydrolysate achieved a biomass concentration of 2.83 g/L consisting of 34% lipids and 26% carbohydrates. These results confirmed that SSB hydrolysate is a promising feedstock for mixotrophic cultivation of *Chlorella* and synthesis of algal bioproducts and biofuels.

## Introduction

Microalgae-based products, such as biofuels, nutraceuticals, pigments, and cosmetics, are viewed as potentially sustainable replacements of fossil- or food-derived products that can reduce carbon intensity and hence greenhouse gas emissions. In recent years, worldwide algae cultivation has expanded significantly at an annual rate of ~ 3%^1^. Still, despite the promise of algal biofuels and bioproducts, algae cultivation is not yet commercially viable due to high capital and operating costs^2^. Algae are generally cultivated in autotrophy (using light and CO_2_) that suffers from low biomass productivity due to self-shading and insufficient CO_2_ gas–liquid exchange in the media^3^. Although CO_2_-rich industrial flue gases can be utilized to enhance biomass productivity, costly infrastructure for their capture and transport is required, while up to 97% of the CO_2_ pumped into open-pond cultivation systems escapes to the atmosphere^3,4^. Furthermore, a techno-economic analysis determined that ~ 65% of the cultivation cost is associated with CO_2_ supply to open ponds^5^. Hence, the use of exogenous carbon sources under heterotrophic (dark) or mixotrophic (light) conditions holds promise as it significantly enhances biomass productivity and product biosynthesis (lipids, proteins, and carbohydrates)^6,7^. Among the cultivation strategies, mixotrophy reportedly augments biomass productivity more than heterotrophy due to simultaneous utilization of the pentose phosphate pathway and photosynthesis^6^. In addition, heterotrophic cultivation of algae is costlier than mixotrophy, since it requires supply of O_2_ during the fermentation and part of the consumed carbon is released by the cells as CO_2_ to the environment^8^.

Despite the aforementioned metabolic advantages, addition of organic carbon to cultivation media significantly increases the cost of algae cultivation. A possible solution is the employment of waste and abundant agricultural residues to generate hydrolysates containing sugars (organic carbon) as a means of significantly reducing the cost and enhancing the sustainability of mixotrophic algae cultivation. To date, several agricultural residues have been reported for cultivation of algae, such as sugarcane bagasse, cassava bagasse, corn stover, and rice straw, resulting in significantly enhanced biomass production and intracellular lipid content^9^. Sweet sorghum bagasse (SSB), a waste biomass residue generated around the world, has not been tested yet for algae cultivation. Sweet sorghum is a promising C4 energy crop with shorter cultivation cycle (3–5 months), higher biomass and sugar yield per hectare than sugar beet, higher resistance to drought and salinity than other sugar crops, and lower fertilizer demand^10,11^. The SSB that remains after sugar extraction consists primarily of cellulose (~ 46%), hemicellulose (~ 26%), and lignin (~ 15%)^12^ making it an excellent source of inexpensive sugars for algae cultivation. Among algal species, *Chlorella* has been extensively investigated for several industrial applications due to its inherent ability to accumulate large amounts of lipids, proteins, and other value-added products^13,14^. *Chlorella* also has robust metabolic capabilities that enable it to thrive under autotrophy, heterotrophy, and mixotrophy, making it a strong candidate for large-scale industrial applications.

The aim of the present study is to establish the potential of SSB hydrolysate for sustainable algal biomass and lipid production through strain selection, process parameter optimization, and cultivation strategy evaluation. A time course investigation of *Chlorella vulgaris* was carried out under all trophic modes (autotrophic, mixotrophic, and heterotrophic) using optimized process parameters to gain insights into physiological alterations at the cellular level. Moreover, cellular carbohydrate and lipid profiling was used to shed light on the metabolic changes occurring in the alga in response to the employed trophic mode. Finally, cultivation in a bench-top PBR was carried out to assess the scalability of SSB hydrolysate for algae production.

## Results and discussion

### Strain selection and Box-Behnken design optimization

**Figure 1:**
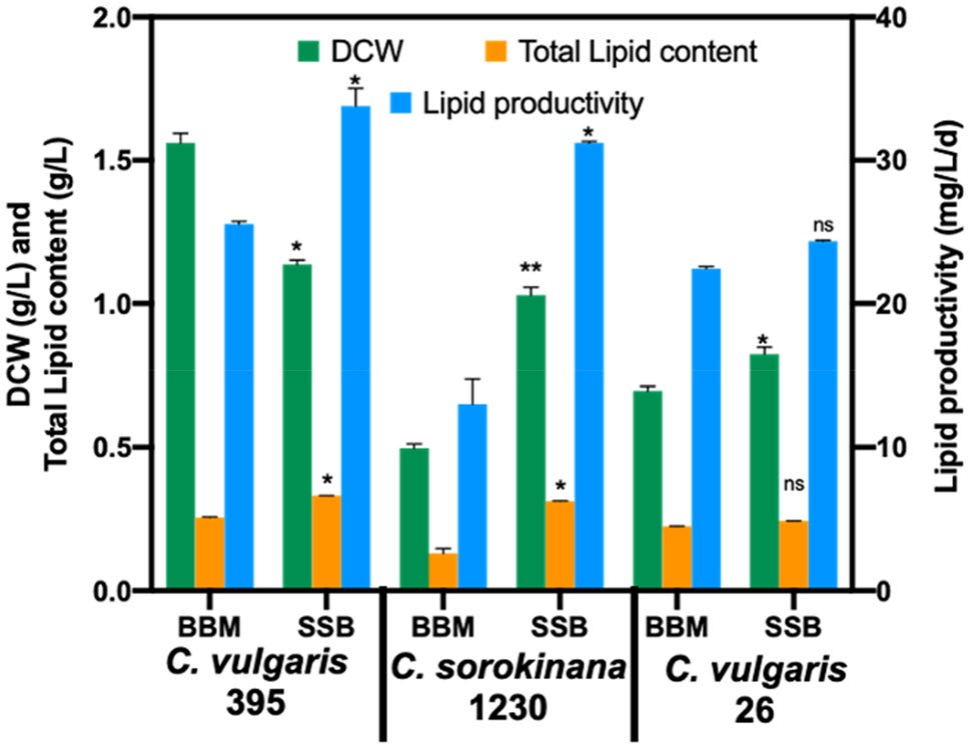
Dry cell weight (DCW), total lipid content, and total lipid productivity of *Chlorella* 395, *Chlorella* 1230, and *Chlorella* 26 on the 10th day of batch cultivation in BBM medium (BBM) vs. SSB hydrolysate (SBB). (*p < 0.05; **p < 0.01; ns: not significant).

Three *Chlorella* strains, *Chlorella vulgaris* UTEX 395 (henceforth referred to as *Chlorella* 395), *Chlorella vulgaris* UTEX 26 (henceforth referred to as *Chlorella* 26), and *Chlorella sorokinana* UTEX 1230 (henceforth referred to as *Chlorella* 1230) reported to achieve high biomass and lipid content^7,15,16^, were cultivated in SSB hydrolysate. The time courses of DCW, lipid content, and lipid productivity of the *Chlorella* strains cultivated in BBM and SSB hydrolysate for 10 days are shown in Fig. 1. All species were able to grow in SSB hydrolysate containing 60 g/L of glucose and 5 g/L of xylose and accumulated ~ twofold higher DCW and lipid content compared to typical rich algae media (BBM). Among the algal strains, *Chlorella* 395 showed the highest lipid productivity (33.8 ± 1.2 mg/L/d) and was thus selected for further studies (Fig. 1). These results are in agreement with previously reported studies, where addition of sugars, particularly glucose, under mixotrophic conditions augmented growth and lipid accumulation^7,15,17^.

Although SSB enhanced lipid productivity in algae compared to BBM, such a high initial glucose concentration (60 g/L) might have caused growth inhibition, since it has been reported that substrate inhibition in *Chlorella* can take place in the presence of 27–40 g/L of glucose^18^. To identify the cultivation conditions that lead to maximum lipid productivity, a Box-Behnken Design (BBD) was utilized in the study. Based on preliminary experiments (not shown), three independent process variables were selected for evaluating *Chlorella* 395 lipid productivity in mixotrophy: inoculum cell density expressed in OD_750_ (at 750 nm), hydrolysate content in the medium expressed in volumetric % , and salinity of the medium expressed in % (g/dL) of exogenously provided sodium chloride, as shown in Table 1. Inoculum density may impact the culture’s adaptation to the cultivation conditions thus affecting biomass productivity, whereas hydrolysate content may have a positive or negative effect on cell growth and lipid production because of various compounds it may contain. Finally, salinity represents a stressor for freshwater algae, like *Chlorella*, which may affect growth and lipid production (Supplementary Table 2).

**Table 1:** Range and coded values for the three independent variables studied in the BBD design of algae cultivation: cell density (OD_750_), hydrolysate content of the medium (%), and medium salinity (%).

		Low, center, and high value		
Parameter	Label	−1	0	1
OD_750_	OD	0.2	0.6	1
SSB hydrolysate (%)	H	25	62.5	100
Salinity (%)	S	0	1.5	3

A second-order polynomial model of the three independent variables was fitted to the experimental data obtained from the BBD with lipid productivity being the dependent variable:

$$\begin{aligned} & {\text{Lipid productivity }}\left( {\text{mg/L/d}} \right) = 115.2\, + \,97.7\left[ {{\text{OD}}_{750} } \right] - 1.534\left[ {\text{Hydrolysate content}} \right] \\ & \quad - 12.47\left[ {{\text{Salinity}}} \right] - 61.9\left[ {{\text{OD}}_{750} } \right]\left[ {{\text{OD}}_{750} } \right]\, + \,0.00768\left[ {\text{Hydrolysate content}} \right]\left[ {\text{Hydrolysate content}} \right] \\ & \quad - 3.77\left[ {{\text{Salinity}}} \right]\left[ {{\text{Salinity}}} \right] - 0.016\left[ {OD_{750} } \right]\left[ {\text{Hydrolysate content}} \right]\, + \,7.31\left[ {OD_{750} } \right]\left[ {{\text{Salinity}}} \right]\, + \,0.0821\left[ {\text{Hydrolysate content}} \right][{\text{Salinity}}] \\ \end{aligned}$$

**Figure 2:**
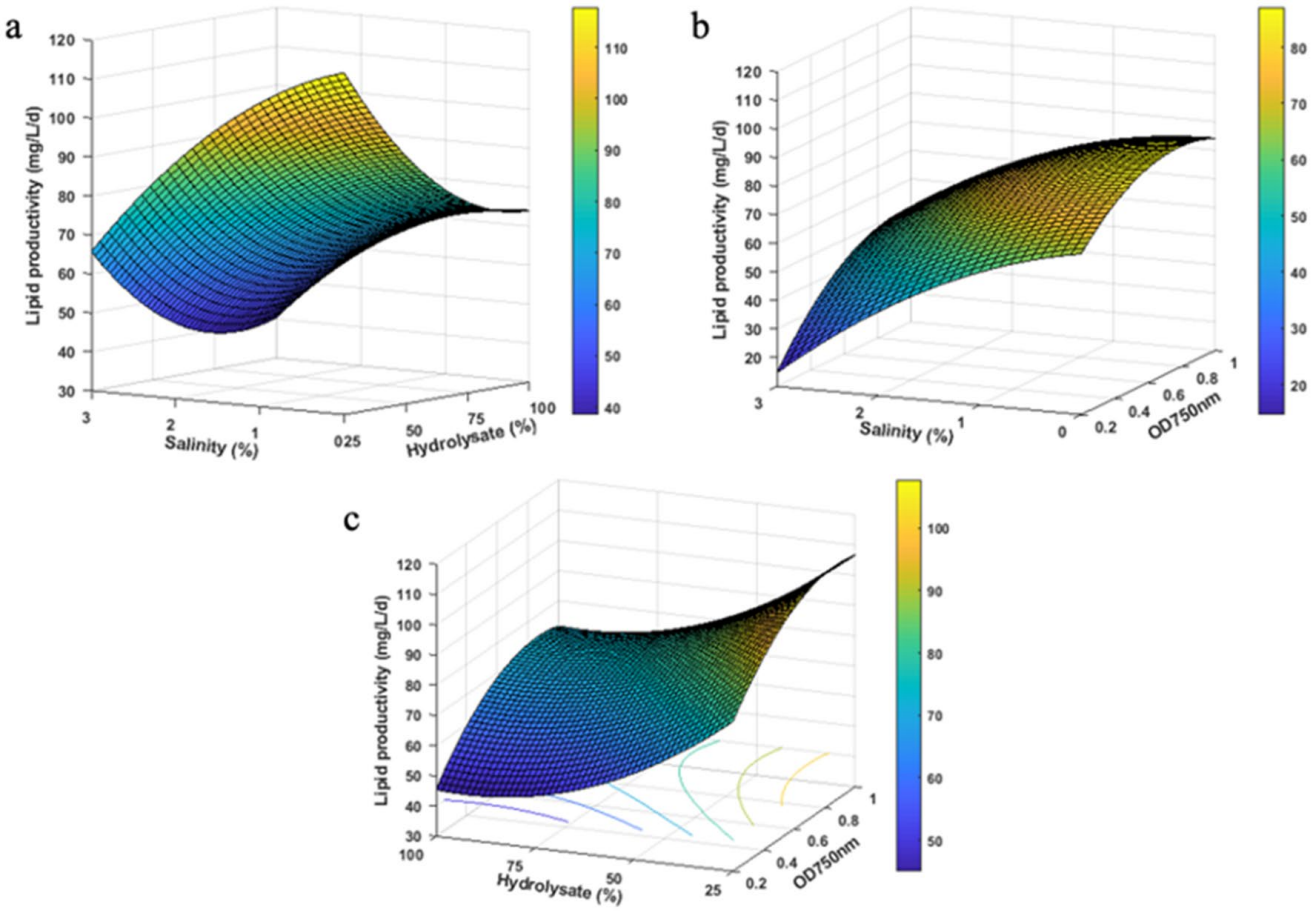
Response surface plots (3D) showing the effect of (**a**) salinity and hydrolysate; (**b**) OD_750_ and salinity; and (**c**) hydrolysate and OD_750_ on lipid productivity of *Chlorella* 395.

A maximum lipid productivity of 119.92 mg/L/D was predicted by the model at OD_750_ = 0.786, hydrolysate content of 25%, and zero salinity (0%). Statistical analysis done with ANOVA, as presented in Supplementary Table 3, yielded an R^2^ value of 85.9% indicating a reasonable agreement between observed and predicted values for the response (lipid productivity). The calculated *F*-value of 13.52 and *p *value of < 0.05 for all three dependent variables indicated that the model was significant. In order to understand the interaction effects between the three variables, 3-D response surface plots of lipid productivity were created for each two-variable combination, while fixing the third variable at its center value (Fig. 2a–c). The plots clearly indicate that lipid productivity was positively correlated to OD_750_ (positive linear coefficient of 13.37), while negatively correlated to both salinity and hydrolysate content with linear coefficients of -21.40 and -17.28, respectively (Supplementary Table 3). Apparently, the higher inoculum cell density facilitates quick adaptation of the algae to the cultivation conditions minimizing the lag phase, while an increase in salinity imposes stress on freshwater algae, like *Chlorella*, leading to a reduced growth rate and lipid production^19^. Similarly, a negative effect on lipid productivity was observed at SSB hydrolysate content beyond 25%, which corresponded to an initial glucose concentration of 15 g/L and xylose concentration of 1.25 g/L. It has been reported that the optimum glucose concentration for *C. vulgaris* ranges between 15 and 25 g/L, and beyond that level the algae do not consume sugar^20,21^. On the other hand, some *C. vulgaris* strains show maximum growth and lipid accumulation in the presence of 40–70 g/L of glucose, indicating that glucose tolerance is a species-specific feature and hence requires optimization for each algae strain^22,23^.

### Effects on physiology of *Chlorella* 395

In order to experimentally validate the model and evaluate the effects of SSB hydrolysate on the physiology of *Chlorella* 395, time course experiments were conducted for 10 days. The model-based optimal conditions of 0.786 inoculum OD_750_, 25% SSB hydrolysate in 75% BBM, and 0% medium salinity were used for cultivating *Chlorella* 395 under both mixotrophy and heterotrophy. Furthermore, cultivation of the alga under autotrophic conditions in BBM and mixotrophic conditions in pure sugars (at the same concentrations of glucose and xylose as in 25% SSB hydrolysate) served as controls. The nitrate and phosphate concentrations in both BBM and pure sugar media were 180 mg/L and 162 mg/L, respectively, whereas in 25% SSB hydrolysate they were 168 mg/L and 2.27 g/L, respectively. The glucose and xylose concentration in pure sugars and SSB hydrolysate were 15 g/L and 1.25 g/L, respectively (Supplementary Table 1).

#### Changes in cell growth

**Figure 3:**
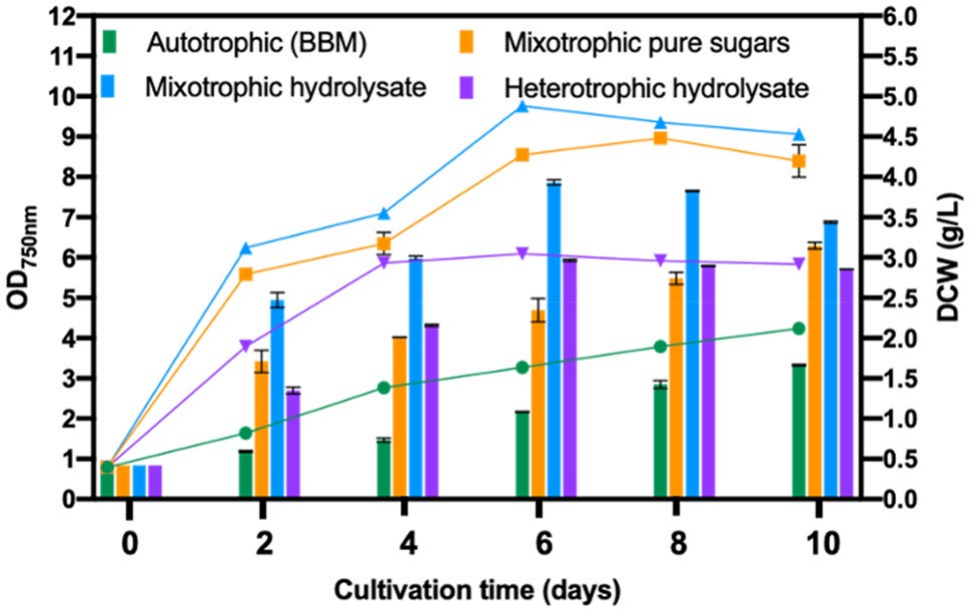
Growth performance in terms of OD_750_ and dry cell weight of *Chlorella* 395 cultivated in various growth media and trophic modes. Lines represent OD_750nm_, whereas bars represent DCW.

The growth curves of the cultures showed that *Chlorella* 395 readily adapted to all conditions without undergoing a lag phase (Fig. 3). However, under mixotrophic and heterotrophic conditions, the cells grew much faster than in autotrophy with exponential growth lasting till the 6th day of cultivation, followed by an early stationary phase on 8th–10th day (Fig. 3). Maximum DCW of 3.44 ± 0.02 g/L and biomass productivity of 0.34 g/L/d were attained under mixotrophy utilizing SSB hydrolysate, closely followed by mixotrophic cultivation in pure sugars (3.15 ± 0.04 g/L), which were 2 and 1.2-fold higher than those in autotrophic and heterotrophic cultivation, respectively (Fig. 3).

It is worth noting that higher growth was recorded using SSB hydrolysate compared to pure sugar media indicating a possible stimulatory effect of metal ions present in the hydrolysate. Previous studies have reported that the presence of Ca^+2^ and phosphorous along with other undefined complex substances, such as amino acids, vitamins, organic acids, and inorganic compounds in biomass hydrolysates enhances the growth rate of *Chlorella*^24,25^. The presence of high amounts of phosphorous (~ 2.3 g/L) in SSB hydrolysate, as a result of its preparation^26^, may have also contributed to the high DCW in hydrolysate as compared to pure sugars. Phosphorous is an essential nutrient for nitrate uptake, energy transfer, and photosynthetic respiration and plays a key role in cell proliferation^27^.

#### Changes in pH, nutrient uptake, and sugar consumption

**Figure 4:**
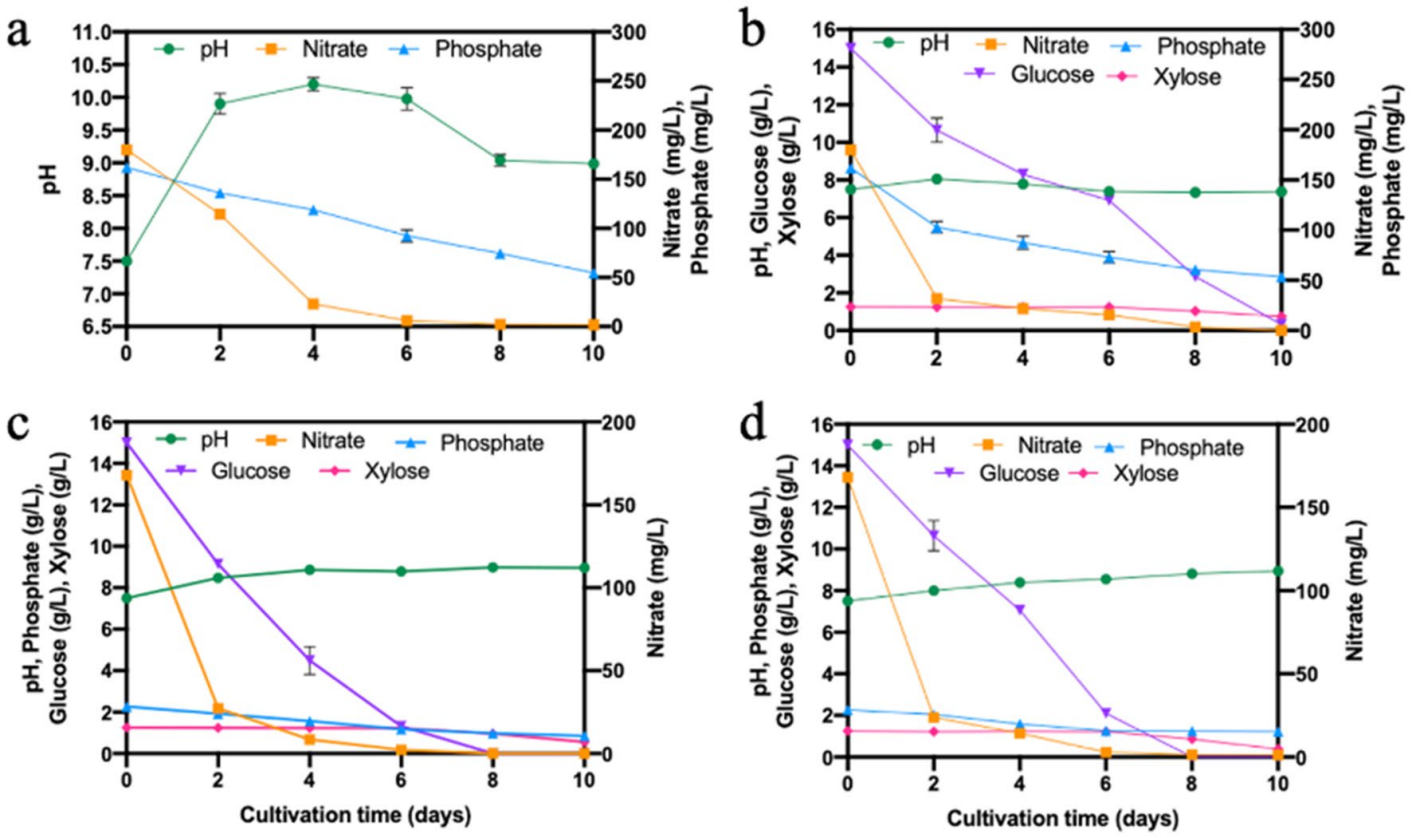
Temporal changes in pH, nitrate, phosphate, glucose, and xylose concentration of the culture media of *Chlorella* 395 cultivated (**a**) autotrophically; (**b**) mixotrophically in pure sugars; **(c)** mixotrophically in SSB hydrolysate; and **(d)** heterotrophically in SSB hydrolysate.

The pH of cultivation media plays an important role in algal growth as it determines the solubility and uptake of nutrients and ideally ranges between 7 and 9 for *Chlorella*^28^. In the present study, for all cultivation runs in flasks the initial pH of the media was set to 7.5 before inoculating *Chlorella* 395 but was not controlled. In autotrophic cultures the pH increased to 9.90 ± 0.16 within just 2 days and then gradually leveled off till the 6th day, followed by a slight decrease from 8th–10th day (Fig. 4a).

Such an increase in pH was due to the fixation of CO_2_ by algae causing generation of OH^-^ ions that are neutralized by H^+^ ions in the media^29,30^. However, when the culture reached early stationary phase with negligible growth and nitrate uptake, the pH dropped due to accumulation of H^+^ ions from the release of organic acids by the cells into the culture media. In contrast, during pure sugar cultivation no significant change in pH was recorded under mixotrophic and heterotrophic conditions as it rose to ~ 8.5 in 2 days and then remained rather stable (Fig. 4b–d). This is due to the fact that both photosynthesis and respiration are simultaneously active, hence CO_2_ and organic carbon are being co-metabolized by the cells leading to insignificant change in the pH^30^. In hydrolysate cultures operated in mixotrophy and heterotrophy, an increase in pH from 7.5 to 9.0 was recorded, which may be explained by the uptake of amino acids present in the SSB hydrolysate^31^.

Nutrient (nitrate, phosphate) and sugar (glucose, xylose) uptake by *Chlorella* 395 cultivated in the various trophic modes is shown in Fig. 4a–d. In autotrophic cultures, a rapid uptake of nitrate was recorded consuming ~ 90% of it within 6 days (exponential phase), followed by a decline in its assimilation during 8th–10th day with ~ 2 mg/L nitrate still remaining in the media at the end (Fig. 4a). In mixotrophic cultures in pure sugars and SSB hydrolysate, ~ 85% of nitrate was consumed within 2 days and was completely exhausted within 6 days, while in heterotrophic cultures nitrate uptake stopped after the 6th day leaving ~ 1.2 mg/L (Fig. 4b–d). Such a high nitrate assimilation was mainly due to the fast growth of the algae in the presence of glucose. Similar results have been reported for *Scenedesmus obliquus*, where the highest assimilation of nitrate was recorded in mixotrophy, followed by heterotrophy and autotrophy^32^. In addition, 10 mg/L of ammonia present in 25% SSB hydrolysate were also completely consumed within 4 days (data not shown) in both the mixotrophic and heterotrophic cultures.

In autotrophic cultures, a gradual increase in phosphate uptake by the algae was recorded with 64% of phosphate being consumed by the end of the cultivation period (Fig. 4a). On the other hand, higher phosphate uptake was recorded in pure sugar cultures (34% uptake in 2 days and 53% uptake in 6 days) with 52 mg/L phosphate still left in the media at the end of cultivation period (Fig. 4b). In the cultures utilizing SSB hydrolysate, since the initial phosphate concentration was very high (2.27 g/L), the uptake was 16% and 12% in 2 days under mixotrophic and heterotrophic conditions, respectively, and increased to 50% and 46%, respectively, by the 6th day (Fig. 4c-d). It has been reported that glucose enhances phosphate assimilation in *C. vulgaris*, as higher biomass production increases nutrient uptake^33^. After the 6th day the heterotrophic cultures did not utilize any more phosphate, while in mixotrophic cultures the phosphate content in the media decreased by an additional 10% by the 10th day (Fig. 4c–d). These observations are in agreement with reports that during active growth (cell division) algae cells assimilate phosphate to synthesize nucleic acids and structural phospholipids, but the uptake gradually decreases as the cells reach stationary phase^34^.

The sugar consumption data clearly indicate that *Chlorella* 395 preferred glucose as carbon source over xylose (Fig. 4b–d). Mixotrophic cultures in SSB hydrolysate consumed glucose more rapidly (~ 90% uptake in 6 days) compared to mixotrophic cultures in pure sugars (~ 53%) and heterotrophic cultures in SSB hydrolysate (~ 86%). Moreover, glucose was completely exhausted by the 8th day in both SSB hydrolysate cultures, whereas some residual glucose (0.25 g/L) remained in pure sugar cultures. The consumption of glucose can be directly related to the highest growth and biomass accumulation observed in mixotrophic cultures utilizing SSB hydrolysate (Fig. 3). Additional inorganic elements present in the hydrolysate may stimulate the uptake of glucose compared to pure sugar media^25^. Glucose transport across the algal membrane is known to occur via an inducible H^+^-hexose symport system encoded by the glucose transporter gene HUP1^35,36^. Glucose is then metabolized via the Embden-Meyerhof pathway and the pentose phosphate pathway (PPP)^37^. Interestingly, xylose assimilation was observed only after glucose was exhausted in all cultures, which occurred during the 8th–10th day (Fig. 4b–d). The hexose transporter reportedly has higher affinity for glucose and shows broad specificity for all aldo- and keto-hexoses, d-glucosamine, and two pentoses (d-arabinose and d-xylose)^38^. It has been reported that the presence of glucose in the growth media induces xylose utilization by microalgae indicating the existence of a distinct xylose metabolic pathway^39^. Moreover, xylose consumption by the microalgae requires light, since its metabolism needs NADPH generated from photosynthesis ^40^. Similar results have been reported for *Chlorella protothecoides*, *Scenedesmus obliquus,* and *C. sorokinanana* ^40–42^.

#### Alterations in biochemical composition and photosynthetic pigments

**Figure 5:**
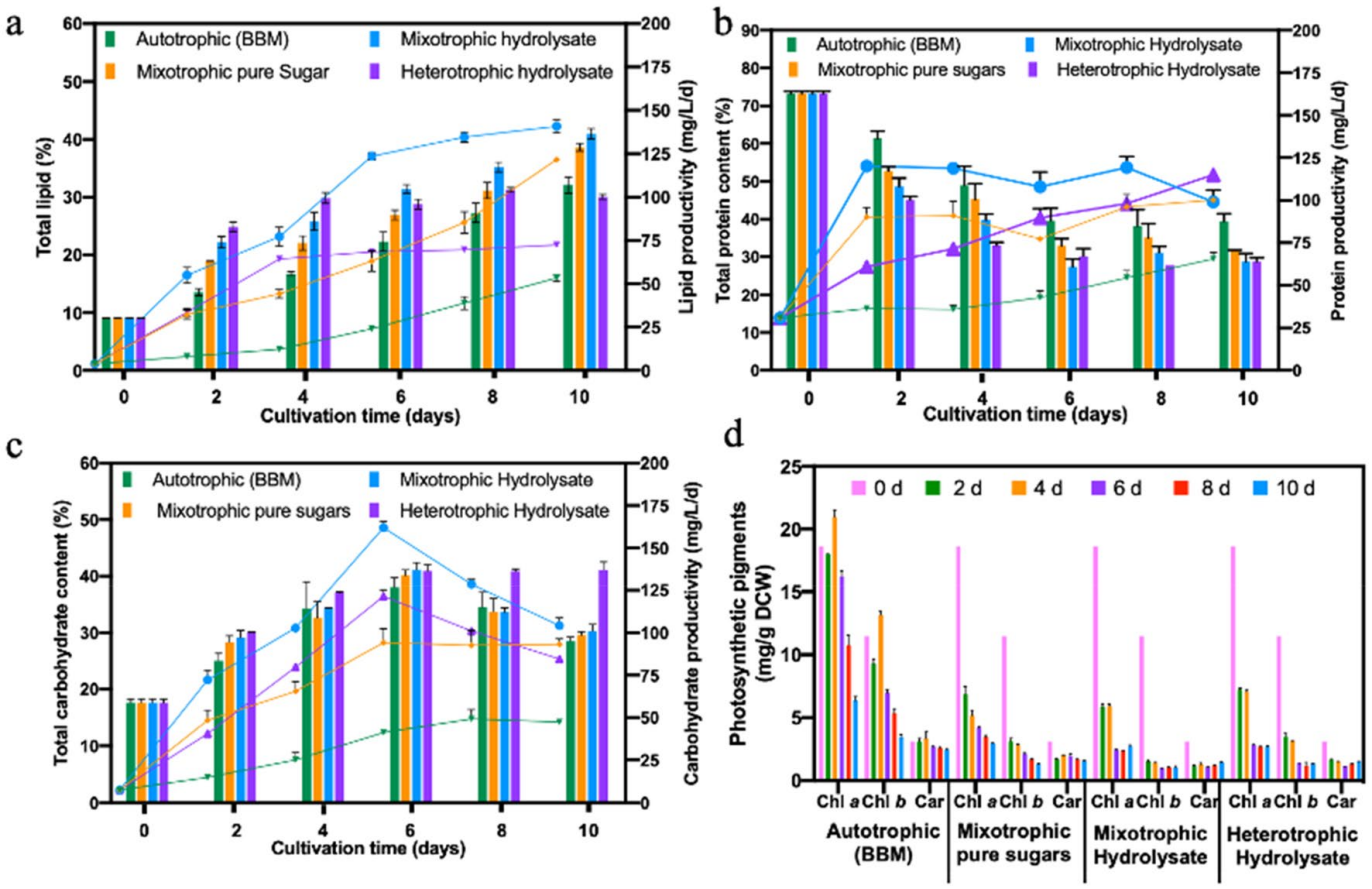
Changes in **(a)** total lipid content and productivity; **(b)** total carbohydrate content and productivity; **(c)** total protein content and productivity; and **(d)** photosynthetic pigment content of *Chlorella* 395 cultivated in various growth media and trophic modes. Lines represent productivity, whereas bars represent cellular content (%).

Depending on growth conditions, algae alter their metabolism to regulate their biochemical composition and photosynthetic pigments. In order to shed light on the metabolic shifts occurring inside *Chlorella* 395, the cells were analyzed for total lipid, carbohydrate, protein, and photosynthetic pigment (chlorophyll *a*, chlorophyll *b*, and carotenoids) content. In all cultures an apparent increase in cellular carbohydrate and total lipid content with a concomitant decrease in protein content was observed, reaching maximum carbohydrate accumulation on the 6th day, whereas the highest total lipid content was reached on the 10th day (Fig. 5a–c).

In mixotrophic and heterotrophic cultures utilizing SSB hydrolysate and pure sugars, the lipid content increased by ~ 13–15% and ~ 10%, respectively, within 2 days, followed by a slower increase of 4–5% through the rest of the cultivation period (Fig. 5a). In contrast, autotrophic cultures showed an increase of just ~ 4% every other day. The fast increase in lipid content in the presence of organic carbon under mixotrophic and heterotrophic conditions has been attributed to the high C/N ratio, which channels the excess carbon flux towards lipogenesis^31^. Moreover, high carbon levels upregulate the expression of genes involved in the Calvin cycle and in glycolysis, thereby driving the synthesis of acetyl-CoA, a fatty acid (lipid) synthesis precursor^43^. Similar results have been reported for *Chlorella* cultivated in food waste hydrolysate^44^. The highest lipid content of 40.92 ± 0.91% and lipid productivity of 141 mg/L/d were recorded in SSB hydrolysate mixotrophic cultures, followed by mixotrophic cultures in pure sugars, heterotrophic cultures in SSB hydrolysate, and autotrophic cultures (Fig. 5a). According to the metabolic mechanism of *Chlorella* 395, the oxidative phase of PPP is inactive under heterotrophic conditions resulting in NADPH limitations^45^. This may be the reason that after the 4th day the lipid content did not increase much under heterotrophic conditions (Fig. 5a).

The highest cellular carbohydrate content (~ 40% of DCW) was recorded in mixotrophic cultures in SSB hydrolysate and pure sugars and was 1.34 and 1.25-fold higher than in heterotrophic and autotrophic cultures (Fig. 5b). The highest carbohydrate productivity of 161 ± 3.6 mg/L/d was attained in mixotrophic cultures utilizing SSB hydrolysate. At the end of the cultivation, a ~ 45% reduction in protein content was recorded in mixotrophic and heterotrophic cultures, while autotrophic cultures showed a ~ 34% decline (Fig. 5c). The observed decrease in protein content was due to depletion of nitrate in the cultivation media during growth. Taken together, these results indicate that *Chlorella* 395 biomass cultivated in SSB hydrolysate under mixotrophy is rich in carbohydrates and lipids and can be potentially utilized for ethanol and biodiesel or renewable diesel production, respectively.

The photosynthetic pigments chlorophyll *a*, chlorophyll *b*, and carotenoids can serve as markers to evaluate the photosynthetic capacity of algal cells. As expected, photoautotrophic cultures had significantly higher total pigment content (12.31 ± 0.44 mg/g DCW) compared to mixotrophic cultures in SSB hydrolysate (5.31 ± 0.15 mg/g DCW) and pure sugars (5.86 ± 0.11 mg/g DCW) and to heterotrophic cultures in SSB hydrolysate (5.59 ± 0.08 mg/g DCW) (Fig. 5d). The low pigment content of algal cells grown in mixotrophy and heterotrophy clearly indicates that the preferred energy source for *Chlorella* 395 under such cultivation conditions is organic carbon, when exogenously provided, compared to CO_2_. Under autotrophy, the chlorophyll *a*, chlorophyll *b*, and carotenoid content increased by 13–16% over the course of the cultivation, peaking on the 4th day and gradually declining by the 10th day (Fig. 5d). Addition of sugars to the cultivation media, irrespective of the trophic mode, resulted in 60–70% decrease in chlorophyll *a* and chlorophyll *b* and 40–45% decrease in carotenoids within 2 days (Fig. 5d). Glucose is known to partially block the transformation of coproporphyrin III, a precursor of chlorophyll *a*^29^. Actually, after sugar depletion under mixotrophic conditions, algae can switch back to photosynthesis by enhancing pigment synthesis^46^. However, in our study, even though glucose was mixotrophically consumed within 6 days, the chlorophyll *a* and chlorophyll *b* contents continued to decrease possibly due to nitrogen depletion. Our findings are in line with previous studies, where a decrease in photosynthetic pigments was recorded under mixotrophy^29,47,48^. Interestingly, carotenoid content remained unchanged in the cells at 1.5–1.8 mg/g DCW (Fig. 5d). Carotenoids are antioxidant molecules that aid in protecting the cells by quenching reactive oxygen species (ROS) generated during nutrient depletion^27^.

#### Distribution of fatty acids and alteration in lipid composition

**Figure 6:**
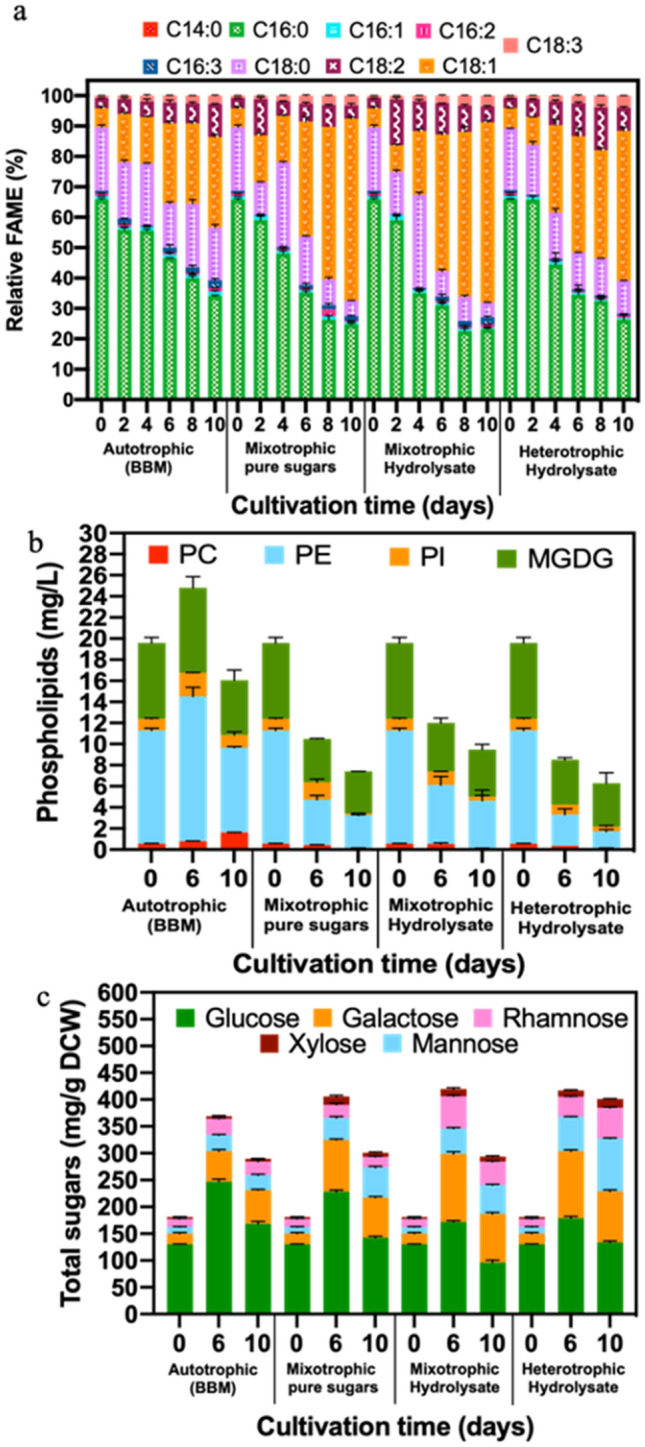
**(a)** Relative changes in FAME composition; **(b)** changes in polar lipid concentration; and **(c)** alterations in cellular carbohydrate content of *Chlorella* 395 cultivated in various growth media and trophic modes.

To understand the metabolic alterations to lipid synthesis in *Chlorella* 395 under various trophic modes, a time course evaluation of FAME profiles was performed. The main fatty acids synthesized in *Chlorella* 395 were palmitic acid (C16:0), stearic acid (C18:0), oleic acid (C18:1), and linoleic acid (C18:2), as shown in Fig. 6a, which that are in line with previous studies^15,28,45^. Initially, a higher proportion of C16:0 (50–55%) was observed by the 6th day in all the trophic modes but it declined afterwards (Fig. 6a). Synthesis of C16:0 is an energy conservation evolutionary mechanism in algae, since its biosynthesis is less energy-intensive and its oxidation releases high amounts of energy^49^. Higher proportions of C18:1 (50–60% of total FAME) and C18:3 (3–4% of total FAME) were observed in cultures cultivated under mixotrophy and heterotrophy as compared to autotrophy (Fig. 6a). Higher C18:1 content is ideal for high-quality biodiesel production^24^. Increased uptake of carbon (organic and inorganic) has been reported to enhance the synthesis of C18:1 in *Chlorella*^24,50^. Furthermore, the addition of organic carbon particularly in mixotrophy resulted in increased monosaturated fatty acid (MUFA) content (60%) compared to heterotrophy (50%) and autotrophy (30%) (Fig. 6a). Biosynthesis of saturated fatty acids (SFA) requires high amounts of photosynthetically produced energy equivalents ATP and NADPH, thus uptake of organic carbon by algae cells tends to favor production of MUFA and polyunsaturated fatty acids (PUFA)^29^. Furthermore, high chlorophyll *a* content in autotrophy has been reported to facilitate high SFA production^51^. A decrease in chlorophyll content under mixotrophy and heterotrophy causes plastid breakage that affects membrane lipid formation^52^. Such a reorganization of cell membrane structure causes recycling of phospholipids, which are mainly composed of PUFA^53^. Overall, the FAME profile of *Chlorella* 395 cultivated in SSB hydrolysate comprised higher SFA and MUFA content, which is reported to be suitable for biodiesel production^54^. Moreover, C16:0, C18:0, and C18:1 fatty acids find applications in various cosmetic formulations^55^.

Polar lipids, such as phospholipids (PL) and galactolipids (GL), are important structural components of the algal cell membrane, which regulates membrane fluidity, selectivity, and stability^56^. The major phospholipids present in *Chlorella* 395 were phosphatidylethanolamine (PE), phosphatidylcholine (PC), and phosphatidylinositol (PI), while monogalactosyldiacylglycerol (MGDG) was the main galactolipid, as depicted in the NMR spectra (Supplementary Fig. 1). The cellular PE, PC, PI, and MGDG contents for each mode of cultivation were analyzed on the 6th and 10th day and were compared to those of cells in the inoculum (day zero sample), as shown in Fig. 6b. Under autotrophic conditions, by the 6th day an increase in all PL and MGDG was recorded with a maximum increase in PI (twofold), followed by PC, PE, and MGDG that showed an increase of 1.3-fold over the day zero control (Fig. 6b). By the 10th day, PC further increased by 2.7-fold, while the PE and MGDG content decreased by 1.5-fold, while no change in PI was recorded. These results indicate strong synthesis of structural polar lipids during the exponential growth phase (0–6th day), while nonpolar TAG lipids increased during the stationary phase (6–10th day), similarly to previous reports for *Chlorella* under nitrogen deprivation^57^. Since the phospholipids PC and PE contain nitrogen in their head groups, a reduced nitrate content in the media most likely will result in a decrease in their synthesis^57^. On the other hand, *Chlorella* 395 cultivated under mixotrophy and heterotrophy showed a much lower PL and MGDG content as compared to autotrophic cultures (Fig. 6b).

In contrast to autotrophy, during mixotrophy in SSB hydrolysate and pure sugars, by the 6th day the levels of PE, PC, PI, and MGDG decreased by 1.2-, 1.9-, 1.2- and 1.7-fold, respectively, while in heterotrophy the decline was 1.5-, 3.0-, 1.2- and 1.7-fold respectively, compared to the zero-day control (Fig. 6b). The phospholipids further declined by the 10th day for both mixotrophic and heterotrophic cultures, although no significant change in the MGDG level was observed (Fig. 6b). Such a decrease in the polar lipids under mixotrophic and heterotrophic cultures indicates that the provided glucose is most likely utilized for TAG synthesis as opposed to structural lipids. This was also reflected in the NMR spectra, where the mixotrophic and heterotrophic cultures showed an increase in the TAG peak compared to the autotrophic cultures (Supplementary Fig. 1). This is the first time the polar lipid distribution in algae is reported under various trophic cultivation conditions.

#### Changes in carbohydrate composition

The algal cell wall is composed of specific carbohydrates that play a vital role in maintaining structural integrity^58^. To decipher the changes in the cell wall of *Chlorella* 395 cultivated in SSB hydrolysate under various trophic modes, cellular monosaccharide composition was analyzed on the 6th day (maximum carbohydrate content) and 10th day (maximum lipid content). The monosaccharide composition of the initial inoculum (day zero sample) showed glucose to be the predominant neutral sugar (72%), followed by galactose (10%), mannose (7%), rhamnose (9%), and xylose (2%) (Fig. 6c). In autotrophic mode, a decrease in glucose content to 66% and 59% of the total carbohydrate content was recorded on the 6th and 10th day, respectively, with a concomitant increase of galactose to 15–22% on the 6th and 10th day, respectively (Fig. 6c). Moreover, a slight increase (2%) in mannose was observed on the 6th day after which it remained constant, while no significant changes with cultivation time were recorded for rhamnose and xylose. An increase in galactose indicates synthesis of structural galactolipids that form the chloroplast membrane of actively growing cells^59^. Furthermore, a decrease in glucose concentration indicates a switch from starch metabolism to lipid synthesis for energy storage purposes after nutrient exhaustion started on the 6th day. Similar results have been reported for *Microctinum* sp. and *C. biconvexa,* when cultivated in vinasse^60^. It is noteworthy that in the presence of organic carbon in the media the galactose content of the cells increased by ~ fivefold between days 0 and 6, followed by a decline of 1.5-fold from the 6th to the 10th day (Fig. 6c). These results indicate channeling of carbon flux towards structural lipids (galactolipids) during the exponential growth phase and then switching to neutral lipid (TAG) synthesis in the early exponential phase. The mannose content increased by ~ 7% from the 6th to the 10th day in mixotrophic and heterotrophic cultures. Since fructose-6-phospate is the common precursor for both glucose and mannose synthesis, such kind of opposite trends between mannose and glucose cellular content are expected^61^. Furthermore, rhamnose (1.5-fold) and xylose (1.6-fold) increased over cultivation time under heterotrophy (Fig. 6c). The presence of mannose has been attributed to the structural mannans in the cell wall or associated glycoproteins in the cell matrix, while xylose has been associated with small amounts of cellulose and hemicellulose in the cell wall of *Chlorella* 395^59^. To the best of our knowledge, the present study reports for the first time on the cellular carbohydrate profile of algae in various trophic cultivation modes.

### Photobioreactor study

To illustrate the scalability of SSB hydrolysate for algae cultivation, *Chlorella* 395 was cultivated in a 2-L bench-top PBR under mixotrophic conditions and was compared to autotrophy. After 7 days of cultivation, mixotrophic cultures utilizing SSB hydrolysate showed maximum DCW of 2.83 ± 0.05 g/L, biomass productivity of 0.41 ± 0.01 g/L/d, lipid productivity of 139 ± 1.43 g/L/d, and carbohydrate productivity of 105.98 ± 7.66 mg/L/d, which were 2.0-, 2.6-, and 1.8-fold higher than those of the autotrophic cultures, respectively (Table 2). These results were comparable with previous reports on *Chlorella* cultivated in other biomass hydrolysates using bioreactors (Table 2).

**Table 2:** Dry cell weight (DCW), carbohydrate content, and lipid content of *Chlorella* reached after cultivation in SSB hydrolysate (this study) and other biomass hydrolysates (literature).

Microalga	Hydrolysate	Cultivation conditions	DCW (g/L)	Carbohydrate (%)	Lipids (%)	Reference
*Chlorella vulgaris*	Food waste (18.23 g/L glucose)	Heterotrophic, 2.5 L bioreactor, 7 days, batch	3.8	28	30	^62^
	Food waste (20 g/L glucose)	Mixotrophic, 2 L reactor, 18 days, 120 μmol photons m^−2^ s^−1^, semi-continuous	6.9	–	26	^44^
	Molasses (50 g/L sucrose)	Mixotrophic, 250 mL glass vessel, 14 days, 200 μmol photons m^−2^ s^−1^, batch	1.2	5.5	7.7	^63^
	Cheese whey (5 g/L glucose and 5 g/L galactose)	Mixotrophic, 0.5 L bioreactor, 8 days, 70 μmol photons m^−2^ s^−1^, batch	3.6	11.5	25	^25^
*Chlorella protothecoides*	Cassava bagasse (20 g/L glucose)	Heterotrophic, 5 L stirred bioreactor, 7 days, batch	6.9	–	35	^64^
*Chlorella vulgaris* 395	Sweet sorghum bagasse (15 g/L glucose and 1.25 g/L xylose)	Mixotrophic, 2 L bioreactor, 7 days, 250 μmol photons m^−2^ s^−1^, batch	2.8	26.1	34.4	This study

The FAME profile mainly comprised C16:0 (31%), C18:1 (43%), and C18:2 (10%) with minor amounts of C14:0, C16:2, C16:3, and C18:3, which is similar to that obtained in the flask cultures (Supplementary Fig. 2). The TECE of the cultures was calculated for both trophic modes at a light intensity of 2.20 kW/m^2^ over 168 h taking into account that the heating value of lipids and glucose are 36.3 kJ/g and 15.64 kJ/g, respectively^65,66^. The TECE of mixotrophic cultures (1.45%) was much higher than in autotrophic cultures (0.62%) indicating better energy conversion in mixotrophy. Even higher TECE may potentially be achieved in mixotrophic cultivation of algae under natural light (outdoors).

## Conclusion

The study demonstrated the feasibility of using hydrolysate of sweet sorghum bagasse, an agricultural residue available around the world, for successfully cultivating *Chlorella vulgaris* at high biomass and lipid productivity in a potentially sustainable way. RSM-based experimentation determined the optimal cell density (OD_750nm_ = 0.786), hydrolysate content (25%), and salinity (0%) to maximize lipid productivity (120 mg/L/D) in the microalga. Mixotrophic cultivation, where the cells used both exogenous sugars and CO_2_, proved to be the most productive cultivation mode, when compared to heterotrophic and photoautotrophic mode, with carbon flux shown to be channeled primarily towards synthesis of nonpolar lipids (TAG). Higher nutrient assimilation (sugar, nitrate, and phosphate) was achieved mixotrophically by *Chlorella* in SSB hydrolysate compared to pure sugars. Moreover, the FAME profile showed higher SFA and MUFA biosynthesis that is advantageous for biofuel and nutraceutical production.

## Materials and methods

### Algae cultivation and SSB hydrolysate preparation

*Chlorella vulgaris* UTEX 395 (henceforth referred to as *Chlorella* 395), *Chlorella vulgaris* UTEX 26 (henceforth referred to as *Chlorella* 26), and *Chlorella sorokinana* UTEX 1230 (henceforth referred to as *Chlorella* 1230) were purchased from the University of Texas at Austin culture collection and grown in Bold’s basal media (BBM; PhytoTech Labs, USA) with the pH set at 7.5 at 25 °C under continuous white light of about 100 µmol/m^2^s in an incubator shaker (Excella E24 from New Brunswick Scientific, Eppendorf, Germany) at 150 rpm. SSB hydrolysate was prepared by pretreating SSB with concentrated phosphoric acid at 50 °C for 43 min at 130 g/L dry biomass followed by enzymatic treatment with cellulase preparation (Cellic Ctech2 cellulase, Novozymes, Denmark) at 50° in a shaking water bath (50 rpm) for 72 h and was stored at 4 °C until use^26^. The generated SSB hydrolysate contained mainly 60 g/L of glucose, 5 g/L of xylose, 132 mg/L of nitrates, 40 mg/L of ammonia, and 8.6 g/L of phosphates (Supplementary Table 1).

### Strain selection

The three *Chlorella* strains were cultivated in BBM or 100% SSB hydrolysate for 10 days in 125-mL Erlenmeyer flasks with working volume of 50 mL starting at an optical density at 750 nm (OD_750_) of 0.2 under the specified growth conditions. Unless otherwise stated, all experiments were conducted using algae inoculum in exponential growth phase. The strain exhibiting the maximum lipid productivity, expressed in mg/L/d, was selected for further studies.

### Response surface methodology for process parameter optimization

Using Box-Behnken Design (BBD), response surface methodology was employed to identify the optimal lipid productivity (mg/L/d) in *Chlorella* using three key cultivation process parameters as independent variables: inoculum cell density (expressed in OD_750_); SSB hydrolysate content in the medium (%), and medium salinity (%). Dilutions of SSB were obtained by adding BBM to 100% SSB hydrolysate. In this design, each parameter was employed at 3 levels, namely low, center, and high, with coded values − 1, 0, and + 1, respectively, as depicted in Table 1. The experimental results obtained from a total of 15 runs replicated twice with 6 center points are summarized in Supplementary Table 2. The results were utilized to fit a second order polynomial model and were statistically analyzed using Minitab software version 19 (Minitab, USA, https://www.minitab.com/en-us/). The derived optimal cultivation process parameters for maximum lipid productivity were employed in the subsequent experimental studies.

### Time course cultivation under various trophic modes

*Chlorella* 395 was cultivated in batch cultures under each of three trophic modes: autotrophic (in BBM), mixotrophic (in SSB hydrolysate or pure sugars), and heterotrophic (in SSB hydrolysate) using the model-based optimal process parameters. The autotrophic and mixotrophic cultures were operated under continuous white light of about 100 µmol/m^2^s, while the heterotrophic culture was operated in the dark. All cultures were housed in an incubator shaker (Excella E24 from New Brunswick Scientific, Eppendorf, Germany) at 150 rpm. To compare the effect of SSB hydrolysate on algal growth and biochemical composition of the cells, a mixture of pure sugars (glucose and xylose) at the same initial concentration as in the SSB hydrolysate was also evaluated. A flask was harvested from each trophic mode and analyzed every two days.

### Analytical methods

#### Growth and dry cell weight determination

Algal growth was monitored by measuring the optical density at 750 nm (OD_750_) of the cell cultures using a DU 730 UV/Vis spectrophotometer (Beckman Coulter, USA). Subsequently, the cells were harvested via centrifugation at 3000*g* for 10 min, washed three times with distilled water to remove media components, and dried in a hot air oven at 50 °C for dry cell weight (DCW) determination^27^. The dried biomass was gravimetrically weighed (Mettler Toledo, USA) and DCW was expressed in g/L. The biomass productivity (g/L/d) was calculated using the following equation:

$$ {\text{Biomass productivity}} = \left( {{\text{Final DCW}} - {\text{Initial DCW}}} \right)/{\text{Cultivation time}} $$

#### Nutrient, sugar, and pH measurement

Culture samples were collected in sterile tubes and filtered through a 0.45 μm filter (Millipore Sigma, USA) before analysis. Colorimetric methods were employed to measure nitrate NO_3_^-^ (NitraVer X Nitrogen-Nitrate Reagent Set), ammonia NH_4_–N (AmVer High Range Ammonia), and phosphate PO_4_^-^ (High Range Total Phosphate) concentrations in the media using kits (Hach, USA). The sugars (glucose and xylose) in the medium were measured using glucose oxidase/peroxidase and enzymatic d-xylose assay kits (Megazyme, Ireland). Medium pH was measured using an Orion 3-Star benchtop pH meter (Thermo Scientific, USA).

#### Biochemical composition and photosynthetic pigment measurement

The biochemical composition (lipids, proteins, and carbohydrates) of algal cells was measured every other day using dry biomass. Total lipids were extracted using the modified Bligh and Dyer method^27,67^. The lipid-extracted biomass was hydrolyzed using 2% H_2_SO_4_ at 121 °C for 15 min in an autoclave and then carbohydrates were quantified using the phenol–sulfuric method^68,69^. Finally, proteins were extracted using a published protocol and quantified by the Bradford assay^70^. The productivities (mg/L/d) of lipids, carbohydrates, and proteins were then calculated using the following equation:

$${\text{Productivity}}\, = \,\left( {{\text{Final content}}{-}{\text{Initial content}}} \right)/{\text{Cultivation time}}$$

The photosynthetic pigment content of *Chlorella* 395 was determined for chlorophyll *a* (Chl *a*), chlorophyll *b* (Chl *b*), and carotenoids. The pigments were extracted using 2 mL of methanol (100%) at 45 °C for 1h^27^. The concentrations of the pigments were then calculated based on absorbance (A) as follows ^71^:

$$ {\text{Chlorophyll}}\,a\left( {{\mu g}/{\text{ml}}} \right) \, = \, 16.72{\text{ A}}_{665.2} {-}9.16{\text{ A}}_{652.4} $$

$$ {\text{Chlorophyll}}\,b\left( {{\mu g}/{\text{ml}}} \right) = 34.09{\text{ A}}_{652.4} {-}15.28{\text{ A}}_{665.2} $$

$$ {\text{Carotenoids }}\left( {{\mu g}/{\text{ml}}} \right) = \left( {1000{\text{ A}}_{470} {-}1.63{\text{ Chl}}\,a{-} 104.9{\text{ Chl}}\,b} \right)/221 $$

These values were then converted to cellular concentrations (mg/g DCW) using the DCW of the algae biomass in the sample.

#### Total lipid and carbohydrate profiling

Extracted total lipids (10 mg) were transesterified in a 5% (v/v) solution of HCl in methanol at 85 °C for 1h^72^. The resulting fatty acid methyl esters (FAME) were extracted with 1 mL hexane and analyzed using GC–MS/MS (Agilent, USA) equipped with a ZB-5HG-G015-11 column (Phenomenex, USA) and tetradecanoic acid methyl ester (C13:0) as internal standard. The employed temperature program was 100 °C for 1 min, then to 200 °C at a rate of 25 °C /min, on hold for 1 min, then to 250 °C at a rate of 5 °C/min, and finally on hold for 7 min, maintaining a 1 mL/min He constant flow^72^. The individual FAME were identified and quantified using the NSIT library search after comparison with FAME standards (Supeclo, Millipore Sigma, USA).

The lipid and carbohydrate profiling were performed on the initial inoculum (day zero sample) and on 6th day and 10th day samples at each of the cultivation trophic modes. For complete cellular lipid profiling, phospholipids and glycolipids were identified using ^1^H NMR (Inova 400 spectrometer, Bruker) with crude lipids (10 mg) suspended in 500 μl deuterated chloroform (CDCl_3_) at 25 °C. The chemical shifts were identified by obtaining spectra for the individual phospholipids phosphatidylcholine (PC), lyso-phosphatidylcholine (lyso-PC), phosphatidylethanolamine (PE), and phosphatidylinositol (PI) (Sigma, USA) and for the galactolipid monogalactosyldiacylglycerol (Avanti Polar Lipids, USA) based on previously published studies^70^.

Cellular carbohydrate profiling was done using 25 mg of hydrolyzed dry algal biomass ^73^. Individual sugars were estimated using Megazyme kits for glucose (GOPOD kit), xylose (d-xylose kit), arabinose and galactose (L-arabinose/d-galactose Assay Kit), rhamnose (L-rhamnose Assay Kit), and mannose (d-mannose/D-fructose/D-glucose Assay kit) and expressed in mg/g DCW.

#### Photobioreactor study

The PBR study under autotrophic and mixotrophic conditions was carried out in a 2-L stirred tank bioreactor (New Brunswick BioFlo 115, Eppendorf, USA) operated at a working volume of 1.5 L, 25 °C, and 200 rpm under continuous white light illumination of about 250 µmol/m^2^s for 7 days. The bioreactor was equipped with temperature, pH, and DO probes and a rotameter for gas flow. The DCW, biochemical composition, and FAME profile were measured at the end of the cultivation period. The total energy conversion efficiency (TECE) was calculated using the following Eq. ^65,66^:

$$ {\text{TECE }}\left( \% \right) = {\text{Heating value of extracted lipids }}\left( {{\text{kJ}}} \right)*100/\left( {{\text{heating value of glucose }}\left( {{\text{kJ}}} \right) + {\text{input light energy }}\left( {{\text{kJ}}} \right)} \right) $$

### Statistical analysis

All experiments were carried out in triplicate (n = 3) and the data are shown as mean ± standard deviation (S.D.). Statistical analysis was done using one-way variance (ANOVA) followed by post-hoc Tukey test (Prism V8, GraphPad Software, USA, https://www.graphpad.com/) with a *p*-value of < 0.05 considered as significant.

## Supplementary Information

Supplementary Information
