# Supplementary material for: Insights into the physiology of Chlorella vulgaris cultivated in sweet sorghum bagasse hydrolysate for sustainable algal biomass and lipid production
Source: Sci Rep. 2021 Mar 24;11:6779. doi: 10.1038/s41598-021-86372-2 (PMC7991646; doi:10.1038/s41598-021-86372-2)
Supplement: Supplementary file 1 — Supplementary Information [file 41598_2021_86372_MOESM1_ESM.docx]

**Supplementary Material**

**Insights into the physiology of *Chlorella vulgaris* cultivated in sweet sorghum bagasse hydrolysate for sustainable biomass and lipid production**

Neha Arora and George P. Philippidis

Patel College of Global Sustainability, University of South Florida

4202 E. Fowler Avenue, Tampa, FL 33620, USA

**^*^Corresponding Author:**

**George P. Philippidis**

Associate Professor

Patel College of Global Sustainability

University of South Florida

4202 E. Fowler Avenue, Tampa, FL 33620, USA

Email: [gphilippidis@usf.edu](mailto:gphilippidis@usf.edu)

**Supplementary Table 1.** Composition of the growth media employed in this study.

The BBM medium had the following composition (g/L): NaNO_3_, 0.25; KH_2_PO_4_, 0.175; K_2_HPO4, 0.075; MgSO_4_.7H_2_O, 0.075; NaCl, 0.025; CaCl_2_.2H_2_O, 0.025; FeSO_4_.7H_2_O, 0.005; EDTA, 0.005; and 1 mL of micronutrient stock containing (g/L): H_3_BO_3_, 2.86; ZnSO_4_.7H_2_O, 0.222; CuSO_4_.5H_2_O, 0.079; MnCl_2_.4H2O, 1.81; Co(NO_3_)_2_.6H_2_O, 0.041 and Na_2_MoO_4_.2H_2_O, 0.390.

| **Component** | **BBM (g/L)** | **Pure sugars (g/L)** | **25 % SSB hydrolysate (g/L)** |
| --- | --- | --- | --- |
| **Glucose** | - | 15 | 15 |
| **Nitrate** | 0.180 | 0.180 | 0.168 |
| **Ammonia** | - | - | 0.04 |
| **Phosphate** | 0.162 | 0.162 | 2.27 |
| **Xylose** | - | 1.25 | 1.25 |

**Supplementary Table 2.** Experimental and predicted responses (calculated from the regression model) of lipid productivity obtained with Box Behnken Design (BBD).

| **Std**  **Order** | **Run**  **Order** | **Pt**  **Type** | **Blocks** | **O.D.750nm** | **Hydrolysate content** | **Salinity** | **Experimental lipid productivity (mg/L/d)** | **Predicted lipid productivity**  **(mg/L/d)** |
| --- | --- | --- | --- | --- | --- | --- | --- | --- |
| 1 | 1 | 2 | 1 | 0.2 | 25 | 1.5 | 76.6 | 76.7 |
| 2 | 2 | 2 | 1 | 1 | 25 | 1.5 | 115 | 103.9 |
| 3 | 3 | 2 | 1 | 0.2 | 100 | 1.5 | 29.5 | 42.7 |
| 4 | 4 | 2 | 1 | 1 | 100 | 1.5 | 68.2 | 68.9 |
| 5 | 5 | 2 | 1 | 0.2 | 62.5 | 0 | 62 | 66.2 |
| 6 | 6 | 2 | 1 | 1 | 62.5 | 0 | 67.8 | 84.2 |
| 7 | 7 | 2 | 1 | 0.2 | 62.5 | 3 | 30.8 | 14.6 |
| 8 | 8 | 2 | 1 | 1 | 62.5 | 3 | 54 | 50.1 |
| 9 | 9 | 2 | 1 | 0.6 | 25 | 0 | 123.2 | 117.8 |
| 10 | 10 | 2 | 1 | 0.6 | 100 | 0 | 90.2 | 74 |
| 11 | 11 | 2 | 1 | 0.6 | 25 | 3 | 49.3 | 65.7 |
| 12 | 12 | 2 | 1 | 0.6 | 100 | 3 | 37 | 40.4 |
| 13 | 13 | 0 | 1 | 0.6 | 62.5 | 1.5 | 73 | 72.2 |
| 14 | 14 | 0 | 1 | 0.6 | 62.5 | 1.5 | 70.8 | 72.2 |
| 15 | 15 | 0 | 1 | 0.6 | 62.5 | 1.5 | 71.7 | 72.2 |
| 16 | 16 | 2 | 1 | 0.2 | 25 | 1.5 | 77 | 76.7 |
| 17 | 17 | 2 | 1 | 1 | 25 | 1.5 | 118.8 | 104 |
| 18 | 18 | 2 | 1 | 0.2 | 100 | 1.5 | 29.9 | 42.7 |
| 19 | 19 | 2 | 1 | 1 | 100 | 1.5 | 69.5 | 68.9 |
| 20 | 20 | 2 | 1 | 0.2 | 62.5 | 0 | 63.8 | 66.2 |
| 21 | 21 | 2 | 1 | 1 | 62.5 | 0 | 68.2 | 84.2 |
| 22 | 22 | 2 | 1 | 0.2 | 62.5 | 3 | 30.8 | 14.6 |
| 23 | 23 | 2 | 1 | 1 | 62.5 | 3 | 52.8 | 50.1 |
| 24 | 24 | 2 | 1 | 0.6 | 25 | 0 | 118.8 | 117.8 |
| 25 | 25 | 2 | 1 | 0.6 | 100 | 0 | 90.2 | 74 |
| 26 | 26 | 2 | 1 | 0.6 | 25 | 3 | 49.7 | 65.7 |
| 27 | 27 | 2 | 1 | 0.6 | 100 | 3 | 37.4 | 40.4 |
| 28 | 28 | 0 | 1 | 0.6 | 62.5 | 1.5 | 70.8 | 72.2 |
| 29 | 29 | 0 | 1 | 0.6 | 62.5 | 1.5 | 72.6 | 72.2 |
| 30 | 30 | 0 | 1 | 0.6 | 62.5 | 1.5 | 73.9 | 72.2 |

**Supplementary Table 3.** Analysis of variance (ANOVA) for the regression model of lipid productivity. DF, SS, MS, F-value, and p-value denote the degrees of freedom, sum of squares, mean of squares, F-statistics, and probability of value, respectively.

| **Source** | **DF** | **Seq SS** | **Contribution** | **Adj SS** | **Adj MS** | **F-Value** | **p-Value** |
| --- | --- | --- | --- | --- | --- | --- | --- |
| Model | 9 | 17552.6 | 85.89% | 17552.6 | 1950.29 | 13.52 | 0.000 |
| Linear | 3 | 14966.0 | 73.23% | 14966.0 | 4988.67 | 34.59 | 0.000 |
| OD_750_ | 1 | 2858.0 | 13.98% | 2858.0 | 2857.97 | 19.82 | 0.000 |
| Hydrolysate content | 1 | 4779.0 | 23.38% | 4779.0 | 4778.96 | 33.14 | 0.000 |
| Salinity | 1 | 7329.1 | 35.86% | 7329.1 | 7329.07 | 50.82 | 0.000 |
| Square | 3 | 2261.6 | 11.07% | 2261.6 | 753.86 | 5.23 | 0.008 |
| OD_750nm_* OD_750nm_ | 1 | 756.4 | 3.70% | 723.8 | 723.77 | 5.02 | 0.037 |
| Hydrolysate content*Hydrolysate content | 1 | 973.5 | 4.76% | 860.5 | 860.54 | 5.97 | 0.024 |
| Salinity*Salinity | 1 | 531.7 | 2.60% | 531.7 | 531.66 | 3.69 | 0.069 |
| 2-Way Interaction | 3 | 325.1 | 1.59% | 325.1 | 108.35 | 0.75 | 0.534 |
| OD_750nm_ *Hydrolysate content | 1 | 0.5 | 0.00% | 0.5 | 0.48 | 0.00 | 0.955 |
| OD_750nm_ *Salinity | 1 | 153.8 | 0.75% | 153.8 | 153.83 | 1.07 | 0.314 |
| Hydrolysate content*Salinity | 1 | 170.8 | 0.84% | 170.8 | 170.76 | 1.18 | 0.289 |
| Error | 20 | 2884.4 | 14.11% | 2884.4 | 144.22 |  |  |
| Lack-of-Fit | 3 | 2856.1 | 13.98% | 2856.1 | 952.05 | 573.61 | 0.000 |
| Pure Error | 17 | 28.2 | 0.14% | 28.2 | 1.66 |  |  |
| Total | 29 | 20437.0 | 100.00% |  |  |  |  |

**Supplementary Figure 1.** ^1^H NMR spectra of *C. vulgaris* 395 lipids **(a)** on the 6^th^ day; and **(b)** on the 10^th^ day of cultivation in various trophic modes.

**(a)**


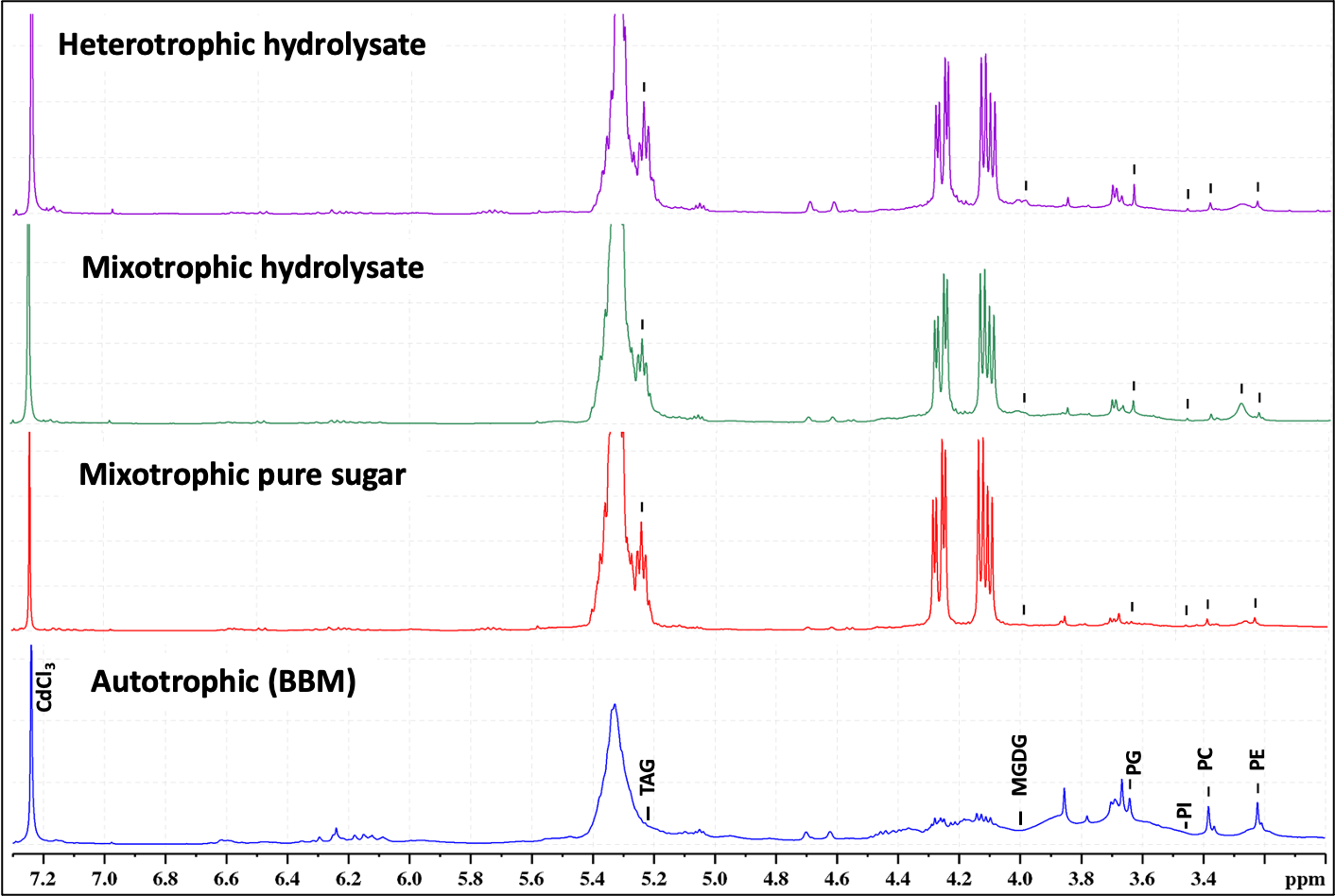


**(b)**

**
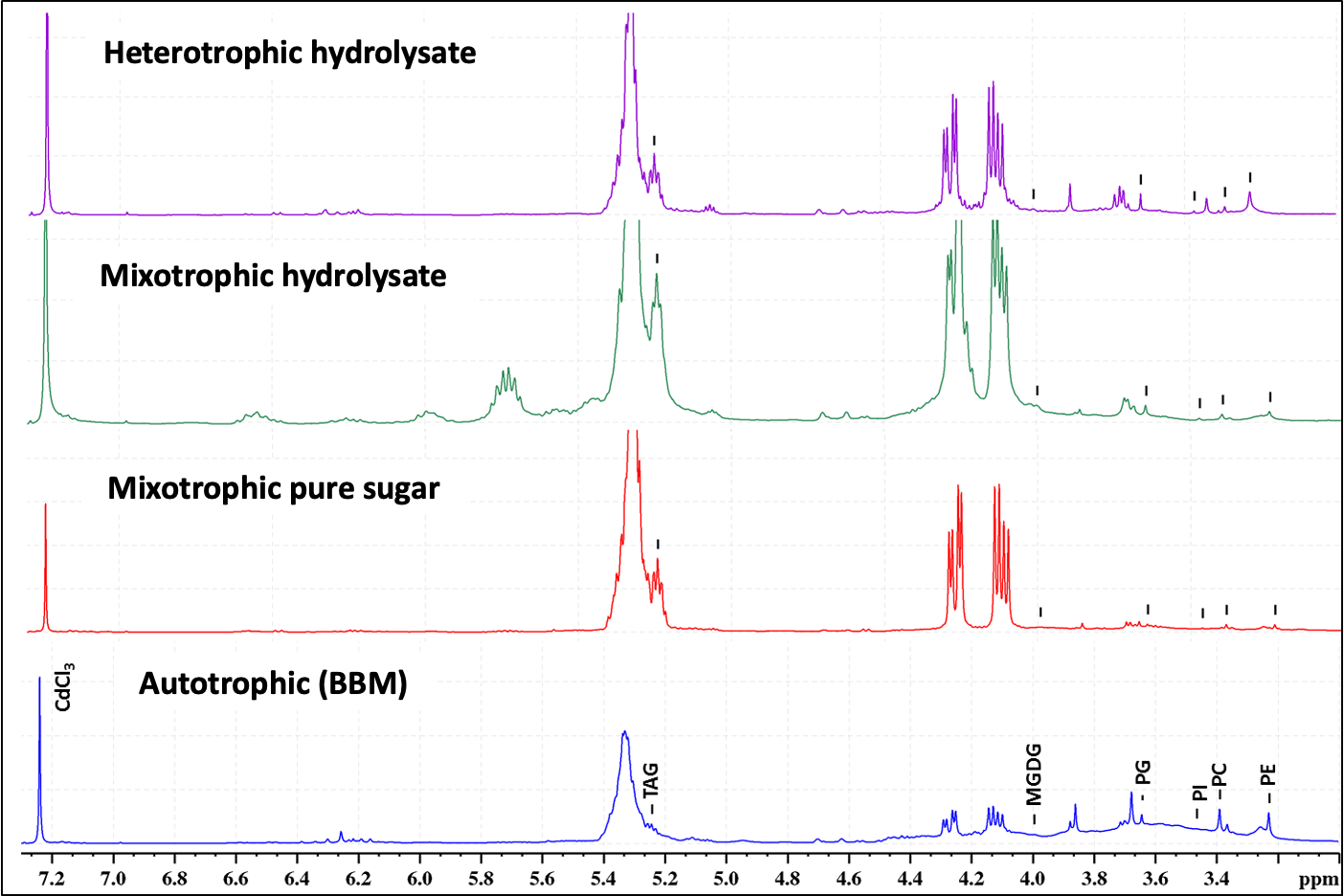
**

**Supplementary Figure 2.** Relative FAME profile of *C. vulgaris* 395 lipids after cultivation in autotrophic mode (BBM) and mixotrophic mode (SSB hydrolysate) in a 2-L photobioreactor.
